# Analysis of Paralleling Limited Capacity Voltage Sources by Projective Geometry Method

**DOI:** 10.1155/2014/359893

**Published:** 2014-02-10

**Authors:** Alexandr Penin

**Affiliations:** “D. Ghitu” Institute of Electronic Engineering and Nanotechnologies, Academy of Sciences of Moldova, Academiei Street 3/3, 2028 Chisinau, Moldova

## Abstract

The droop current-sharing method for voltage sources of a limited capacity is considered. Influence of equalizing resistors and load resistor is investigated on uniform distribution of relative values of currents when the actual loading corresponds to the capacity of a concrete source. Novel concepts for quantitative representation of operating regimes of sources are entered with use of projective geometry method.

## 1. Introduction

The paralleling of lower-power voltage sources (converter modules) offers well-known advantages over a single, high power source. The basic problem of such power supply system is the load-current sharing among the paralleled modules. Various approaches of current distribution are known [1]. In the most simple droop method the equalizing resistors are used [2–4], including lossless passive elements [5]. Usually, equality of module parameters is provided, that is, open circuit voltages and internal resistance. Therefore, the distribution of currents means the equality of these currents.

On the other hand, scatter of module parameters, possible cases of use of primary voltage sources (further-sources) with different capacity, determines the nonuniformity distribution of currents. Therefore, it is natural to understand the uniform loading of sources in the relative sense when the actual loading corresponds to the capacity of the source.

The analysis of such power supply system by the method of projective geometry has led to introduction of some concepts for quantitative representation of operating regimes [6]. In the present paper previously elaborated approach is developed.

## 2. Analysis of the Paralleling of Voltage Sources

**Figure 1:**
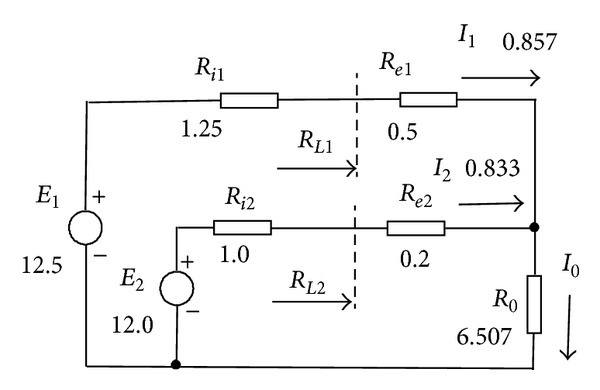
Paralleling of voltage sources.

Let us consider voltage sources *E*_1_, *E*_2_, presented in Figure 1. Resistors *R*_*i*1_, *R*_*i*2_ are internal resistance of these voltage sources; equalizing resistors *R*_*e*1_, *R*_*e*2_ provide current distribution for the given load resistance *R*_0_.

A circuit in Figure 1 is described by the following system of the equations:

(1) $$\begin{array}{c} E_{1}=I_{1}R_{i1}+I_{1}R_{e1}+V_{0},\\ E_{2}=I_{2}R_{i2}+I_{2}R_{e2}+V_{0},\\ V_{0}=I_{0}R_{0}=\left(I_{1}+I_{2}\right)R_{0}. \end{array}$$

The variants of normalized parameters of a loading regime of the first source (used in different areas of electrical engineering, radio engineering, and power) look like

(2) $$\begin{array}{c} m_{1}=\frac{R_{L1}}{R_{i1}}=\frac{I_{1}^{M}}{I_{1}}-1,\;\;J_{1}=\frac{I_{1}^{}}{I_{1}^{M}}. \end{array}$$

Here the maximum current of a source corresponds to the short circuit current:

(3) $$\begin{array}{c} I_{1}^{M}=\frac{E_{1}}{R_{i1}}. \end{array}$$

Also, such relationships can be rewritten as

(4) $$\begin{array}{c} \frac{R_{L1}-R_{i1}}{R_{L1}+R_{i1}},\;\;\frac{I_{1}^{M}-I_{1}}{I_{1}^{M}+I_{1}},\;\;\frac{I_{1}^{M}}{I_{1}} \end{array}$$

and so on.

Let us note essential feature-all of these equations which represent fractionally linear expressions. Moreover, they can be interpreted as projective transformations (transformations in projective geometry) which possess an invariant. Therefore, all above mentioned expressions are equivalent. Further we will use expression (2).

Similarly consideration can be done for the second source:

(5) $$\begin{array}{c} m_{2}=\frac{R_{L2}}{R_{i2}}=\frac{I_{2}^{M}}{I_{2}}-1,\;\;J_{2}=\frac{I_{2}^{}}{I_{2}^{M}}. \end{array}$$

Let us write expressions which associate parameters of source loading in the form *m*_2_(*m*_1_), *J*_2_(*J*_1_). From (1) follows

(6) $$\begin{array}{c} E_{1}-E_{2}=I_{1}\left(R_{i1}+R_{e1}\right)-I_{2}\left(R_{i2}+R_{e2}\right). \end{array}$$

Keeping in mind that

(7) $$\begin{array}{c} I_{1}=\frac{I_{1}^{M}}{m_{1}+1}=\frac{E_{1}}{\left(m_{1}+1\right)R_{i1}},\;\;I_{2}=\frac{E_{2}}{\left(m_{2}+1\right)R_{i2}}, \end{array}$$

we obtain

(8) m2=(−(E1E1−E2+E2E1−E2Re2Ri2)m1   +(E1E1−E2Re1Ri1−E2E1−E2Re2Ri2)) ×(m1−(E2E1−E2+E1E1−E2Re1Ri1))−1=−am1+(d−a+1)m1−d,

(9) $$\begin{array}{c} J_{2}=\frac{d+1}{a-1}J_{1}-\frac{1}{a-1}. \end{array}$$

**Figure 2:**
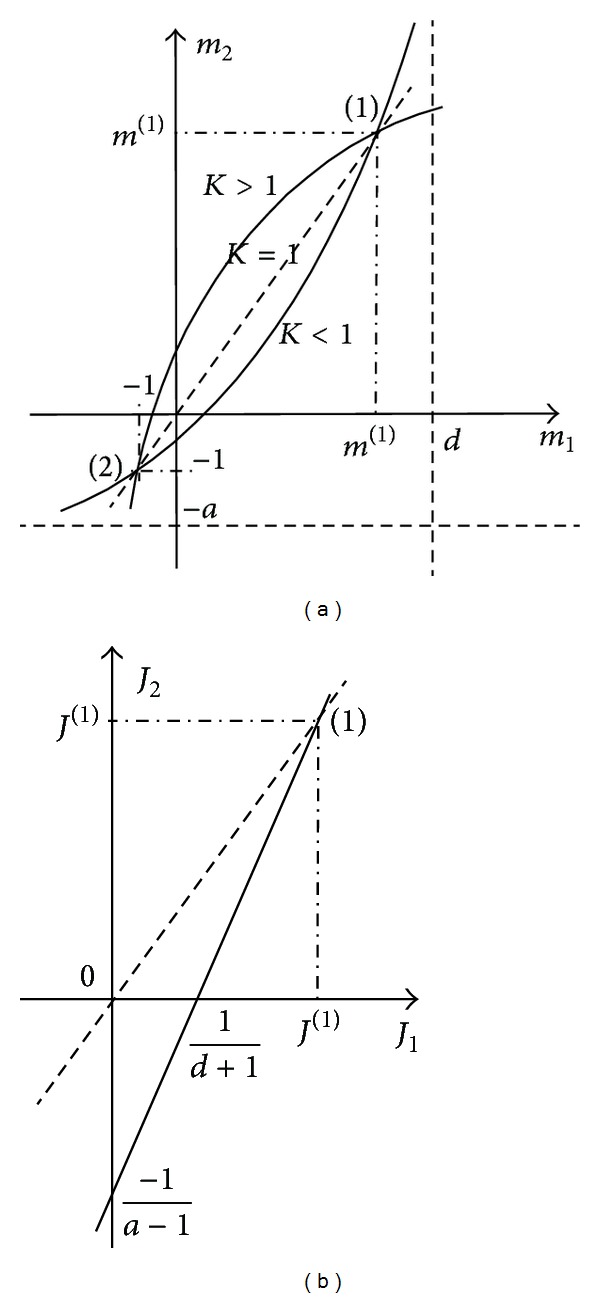
Correlated changes of source loading parameters: (a) is as a family of hyperboles *m*_2_(*m*_1_) for various values of nonuniformity factor of loading *K*; (b) is as a straight line *J*_2_(*J*_1_).

The plots of these dependences are presented in Figure 2.

Expression (8) corresponds to a hyperbole, and (9) corresponds to a straight line. The desirable operating regime corresponds to straight lines on these plots, *m*_2_ = *m*_1_, *J*_2_ = *J*_1_.

**Figure 3:**
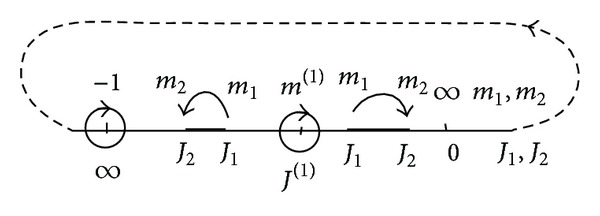
Display of projective transformation of points *m*_1_ → *m*_2_, *J*_1_ → *J*_2_.

The crossing of this straight line with the hyperbole plot gives the two points of equal loading of sources, *m* ^(1)^, *m* ^(2)^. The working area (when load consumes energy) corresponds to the first point *m* ^(1)^. The second point corresponds to the condition when the voltage sources relatively equally consume energy. The points of equal loading are fixed points of projective transformation *m*_1_ → *m*_2_, *J*_1_ → *J*_2_, as it is shown in Figure 3.

In this case, expression (8) leads to a quadratic one:

(10) $$\begin{array}{c} m^{2}-\left(d-a\right)m-\left(d-a+1\right)=0. \end{array}$$

Solution gives the following two roots:

(11) $$\begin{array}{c} m^{\left(1\right)}=d-a+1,\;\;m^{\left(2\right)}=-1, \end{array}$$

that corresponds, also, to the two fixed points of transformation (9):

(12) $$\begin{array}{c} J^{\left(1\right)}=\frac{1}{d-a+2},\;\;J^{\left(2\right)}=\infty . \end{array}$$

For the second fixed point, the currents *I*_2_, *I*_1_ → *∞*. Though this case physically is not feasible, its mathematical description allows entering some necessary characteristics of a circuit.

Let us consider in detail a geometrical interpretation of the transformation (8) for different initial values of quantities *m*_1_, *m*_2_ at loading change. These quantities define a line segment; its length (in the usual sense of Euclidean geometry) or degree of difference of source loading is decreased at its approach to the fixed points. It is obvious that this length for the different circuits will be various.

Thus, it is possible to enter two concepts. One of them defines a circuit: how much the loadings of sources can differ. The second concept defines deviations of actual loadings from a fixed point in the relative form. In this case, it is possible to compare running regimes of the different circuits.

For introduction of such characteristics we use a number of concepts of projective geometry [7, 8], applied in the electric circuit theory [9, 10]. The characteristic of a relative positioning of two points, concerning chosen points (as the special case, it is the fixed points  *m* ^(1)^, *m* ^(2)^), is the cross ratio of these four points:

(13) $$\begin{array}{c} \left(\begin{array}{cccc} m^{\left(2\right)} & m_{2} & m_{1} & m^{\left(1\right)} \end{array}\right)=\frac{m_{2}-m^{\left(2\right)}}{m_{2}-m^{\left(1\right)}}÷\frac{m_{1}-m^{\left(2\right)}}{m_{1}-m^{\left(1\right)}}, \end{array}$$

where the points *m* ^(2)^, *m* ^(1)^ are extreme or base ones. A cross ratio is generalization of a usual proportion. Also it is known that the cross ratio concerning the fixed points does not depend on values of running points *m*_1_, *m*_2_. Therefore, we accept simplification of calculations *m*_1_ = *∞*. Then

(14) (m(2)m2(∞)∞m(1))  =m2(∞)−m(2)m2(∞)−m(1)=a−1d+1=K,K=E2E1·1+Re2/Ri21+Re1/Ri1.

The obtained expression is defined by circuit parameters only. This expression characterizes the ability of a circuit to equal loading of sources that corresponds to the first entered concept. We name this expression as the *factor* *K* *of nonuniformity of loading*.

Obviously, if *m*_2_ → *m*_1_, then *K* → 1 for the given circuit. Generally, the factor of nonuniformity of loading *K* ≠ 1. Equation (13), taking into account (14), allows expressing dependence *m*_2_(*m*_1_), using only the two parameters of a circuit, such as *m* ^(1)^ and *K*.

Let us present (13) as

(15) $$\begin{array}{c} K=\frac{m_{2}+1}{m_{2}-m^{\left(1\right)}}÷\frac{m_{1}+1}{m_{1}-m^{\left(1\right)}}. \end{array}$$

From here

(16) m2=−((1+Km(1))/(1−K))m1+m(1)m1−(K+m(1))/(1−K)=−(1+Km(1))m1+(1−K)m(1)(1−K)m1−(K+m(1)).

The dependence (16) for different values of *K* is presented in Figure 2. For values *K* ≠ 1 a bunch of hyperboles is obtained, which degenerates in a straight line *m*_2_ = *m*_1_ if *K* = 1.

Let us analyze expression (14). Consider *E*_1_ = *E*_2_. Then

(17) $$\begin{array}{c} K=\frac{1+R_{e2}/R_{i2}}{1+R_{e1}/R_{i1}}. \end{array}$$

The condition *K* = 1 leads to the following equality:

(18) $$\begin{array}{c} \frac{R_{e2}}{R_{i2}}=\frac{R_{e1}}{R_{i1}}. \end{array}$$

Therefore, it is quite possible to put *R*_*e*2_ = *R*_*e*1_ = 0. Thus, if voltage sources have identical open circuit voltages, they are equally loaded, and it is independent on their capacity. Generally, expression (14) allows comparing the factors of nonuniformity of loading of different circuits to determine the values of equalizing resistors for necessary value of this factor.

## 3. Comparison of Loading Regime of Different Circuits

Let us obtain normalized representation of the dependence *m*_2_(*m*_1_). For this purpose, we will consider the cross ratios for quantities (or variables) *m*_1_ and *m*_2_, using their conformity, according to transformation (8). Therefore, the cross ratios are equal among themselves:

(19) $$\begin{array}{c} \left(\begin{array}{cccc} -1 & m_{1} & m^{\left(1\right)} & d \end{array}\right)=\left(\begin{array}{cccc} -1 & m_{2} & m^{\left(1\right)} & \infty \end{array}\right), \end{array}$$

where *d* = (*K* + *m* ^(1)^)/(1 − *K*) according to (16).

The cross ratio is a relative expression and gives necessary normalizing of variables. Therefore, any variety of relative expressions for variables *m*_1_ and *m*_2_ is excluded.

Let us present each cross ratio as

(20) (−1m1m(1)d)=m1+1m1−((K+m(1))/(1−K)) ÷m(1)+1m(1)−((K+m(1))/(1−K))=m1+1m1−((K+m(1))/(1−K))·−K1−K,(−1m2m(1)∞) =m2+1m2−∞÷m(1)+1m(1)−∞=m2+1m(1)+1.

Therefore, we have

(21) $$\begin{array}{c} \frac{m_{2}+1}{m^{\left(1\right)}+1}=\frac{m_{1}+1}{m_{1}-\left(\left(K+m^{\left(1\right)}\right)/\left(1-K\right)\right)}\cdot \frac{-K}{1-K}. \end{array}$$

The left side of this expression represents a normalized value and prompts how to write the similar value in the right side. Therefore,

(22) $$\begin{array}{c} \frac{m_{2}+1}{m^{\left(1\right)}+1}=\frac{\left(-K/\left(1-K\right)\right)\left(\left(m_{1}+1\right)/\left(m^{\left(1\right)}+1\right)\right)}{\left(\left(m_{1}+1\right)/\left(m^{\left(1\right)}+1\right)\right)-\left(1/\left(1-K\right)\right)}. \end{array}$$

Similarly, we have by (9)

(23) $$\begin{array}{c} \frac{J_{2}}{J^{\left(1\right)}}=\frac{J_{1}}{J^{\left(1\right)}K}-\frac{1-K}{K}. \end{array}$$

**Figure 4:**
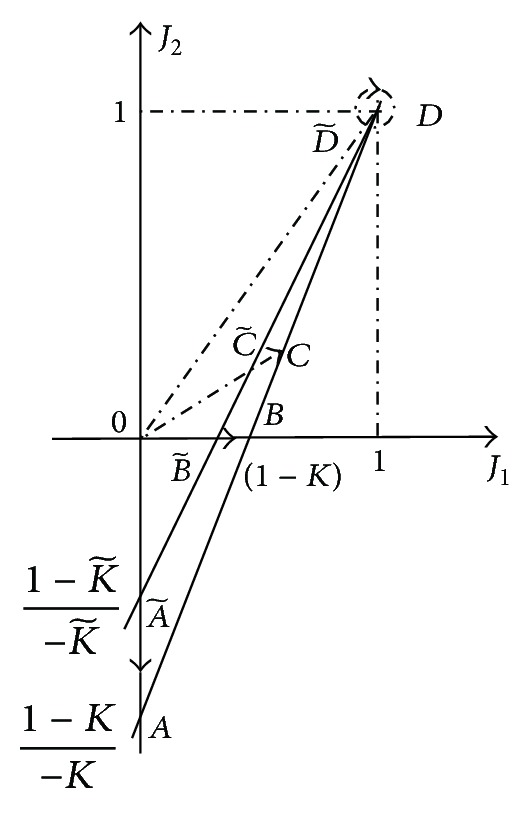
Comparison of loading regime of two different circuits.

It should be noted that expressions (22), (23) certainly set a deviation of running parameters of loading from the equal loading regime in form of normalized values. But, it is not enough for comparison of deviations for circuits with different values of parameter *K*. As an example of the most simple relation (23) we will show why it turns out so. Let us consider two circuits with different values of parameters *K*, $\tilde{K}$ but with the identical value *J* ^(1)^ = 1. Characteristics of the circuits in the form of straight lines are presented in Figure 4.

Loading regimes may be considered identical if conformity of characteristic regime points takes place (shown by arrows in Figure 4) at change of the load. It follows from similarity principles [11]. Then, the projective transformation takes place and it is set by the center at the point 0 and by three pairs of characteristic regime points: *A*, *B*, *D* and $\tilde{A}$, $\tilde{B}$, $\tilde{D}$. The points *D*, $\tilde{D}$ coincide among themselves and correspond to the fixed point *J* ^(1)^. The point of a running regime *C* should correspond to the point $\tilde{C}$. For such projective transformation, the invariant in the form of a cross ratio is carried out; $\left(A\;\;C\;\;D\;\;B)=(\tilde{A}\;\;\tilde{C}\;\;\tilde{D}\;\;\tilde{B}\right)$. Here, the points *A*, *B* and $\tilde{A}$, $\tilde{B}$ are base, and the points *D*, $\tilde{D}$ are unit ones.

Therefore, such cross ratio can be accepted as an equal deviation of the running points *C*, $\tilde{C}$ from the unit points. Further, we display the points *A*, *C*, *D*, *B* on the axis of current *J*_1_. Then, we obtain the deviation for the first source:

(24) Δ1=(0J1J(1)(1−K)J(1))=J1J1−(1−K)J(1)·K,

by another form:

(25) Δ1=(0J1J(1)1d+1)=J1J1−1/(d+1)·a−1d+1=(a−1)J1J1(d+1)−1.

The deviation for the second source is expressed similarly

(26) $$\begin{array}{c} \Delta_{2}=\left(\begin{array}{cccc} 0 & J_{2} & J^{\left(1\right)} & \frac{1}{d+1} \end{array}\right)=\frac{J_{2}}{J_{2}-1/\left(d+1\right)}\cdot \frac{a-1}{d+1}. \end{array}$$

Thus, the deviations include parameters of a circuit and are not simply the normalized values  *J*_1_/*J* ^(1)^, *J*_2_/*J* ^(1)^.

Taking into account the conformity between various definitions of parameters of loading regime, the deviation is expressed in the invariant form through the corresponding cross ratio for the variable *m*_1_:

(27) Δ1=(0J1J(1)1d+1)=(∞m1m(1)d)=m(1)−dm1−d=1−am1−d.

**Figure 5:**
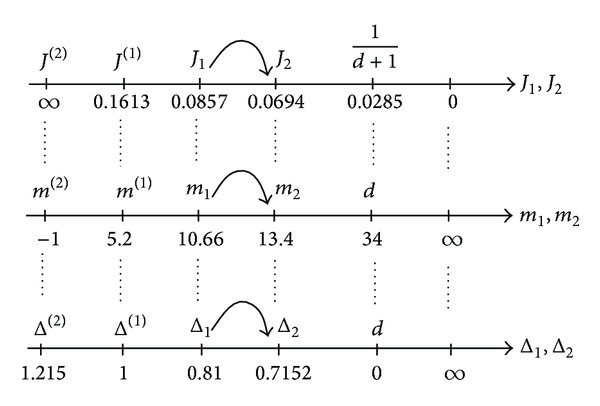
Deviations in the characteristic points.

The values of the deviations in the characteristic points are presented in Figure 5.

In particular, the deviation Δ^(2)^ for the second fixed point, *m* ^(2)^ = −1, is equal to parameter *K*:

(28) $$\begin{array}{c} \Delta^{\left(2\right)}=\frac{a-1}{d+1}=K. \end{array}$$

*Example.* We use the specific values of elements in Figure 1 and dimensions of its values are not indicated for simplifying the record.

 Maximum source currents by (3):  *I*_1_^*M*^ = 10,  *I*_2_^*M*^ = 12.
 Parameters of the loading regimes by (2): *m*_1_ = 10.66, *J*_1_ = 0.0857; *m*_2_ = 13.4, *J*_2_ = 0.0694.
 Expressions of the source loading regimes by (8), (9):
  (29) $$\begin{array}{c} m_{2}=\frac{-29.8m_{1}+5.2}{m_{1}-34},\;\;J_{2}=1.215J_{1}-0.0347. \end{array}$$
 First fixed points by (11), (12): *m* ^(1)^ = 5.2, *J* ^(1)^ = 0.1613.
 Factor of nonuniformity of loading by (14): *K* = 0.8228.
 Deviations for the sources by (25), (27): Δ_1_ = 1.2343 = 1/0.8101, Δ_2_ = 1.398645 = 1/0.715.
 Deviation by (28): Δ^(2)^ = 0.8228.
 All values are presented in Figure 5.

## 4. Conclusions

1. The concept of a nonuniformity factor for voltage source loading which quantitatively characterizes the ability of a circuit for the equal loading of sources is proposed. This factor allows comparing the different circuits.
2. The concept of deviation of running regime from the regime of equal loading in a relative form for a voltage source is entered. It allows comparing the deviations of regime of the sources, being the part of single or different supply systems.
3. Geometrical interpretation grounds the introduction and definition of proposed concepts.
